# A Comprehensive Insight into Māmaki (*Pipturus albidus*): Its Ethnomedicinal Heritage, Human Health Research, and Phytochemical Properties

**DOI:** 10.3390/plants12162924

**Published:** 2023-08-12

**Authors:** Grant Koher, Ajmal Khan, Gabriel Suarez-vega, Pornphimon Meesakul, Ann-Janin Bacani, Tomomi Kohno, Xuewei Zhu, Ki Hyun Kim, Shugeng Cao, Zhenquan Jia

**Affiliations:** 1 Department of Biology, University of North Carolina at Greensboro, Greensboro, NC 27410, USA; gfkoher@uncg.edu (G.K.); a_khan10@uncg.edu (A.K.); g_suarez@uncg.edu (G.S.-v.); 2 Department of Pharmaceutical Sciences, Daniel K. Inouye College of Pharmacy, University of Hawai’i at Hilo, Hilo, HI 96720, USA; pmeesak@hawaii.edu (P.M.); ajbacani@hawaii.edu (A.-J.B.); tkohno@hawaii.edu (T.K.); 3 Department of Medicine, Wake Forest University School of Medicine, Winston Salem, NC 27101, USA; xwzhu@wakehealth.edu; 4 School of Pharmacy, Sungkyunkwan University, Suwon 16419, Republic of Korea; khkim83@skku.edu

**Keywords:** māmaki, *Pipturus albidus*, biological activities, therapeutic properties, human health, phytochemical

## Abstract

In Hawaii, the plants *P. albidus*, *P. forbesii*, *P. kauaiensis*, and *P. ruber* are collectively known as māmaki in ethnomedicine, where *P. albidus* predominates. Farmed māmaki is becoming increasingly popular in Hawaii and the United States. Māmaki teas (such as bottled Shaka tea) are the dominant product. Historically, māmaki has been utilized for its medicinal properties, promoting well-being and good health through consuming tea made from its leaves, ingesting its fruit, and incorporating it into ointments. Māmaki holds cultural significance among Native Hawaiians and is widely used in ethnic medicine, having been incorporated into traditional practices for centuries. However, the scientific mechanisms behind its effects remain unclear. This review consolidates current knowledge of māmaki, shedding light on its potential therapeutic properties, physical properties, nutritional and mineral composition, and active phytochemicals. We also highlight recent research advances in māmaki’s antibacterial, anti-viral, chemopreventive, anti-inflammatory, and antioxidant activities. Additionally, we discuss future prospects in this field.

## 1. Introduction

**Figure 1:**
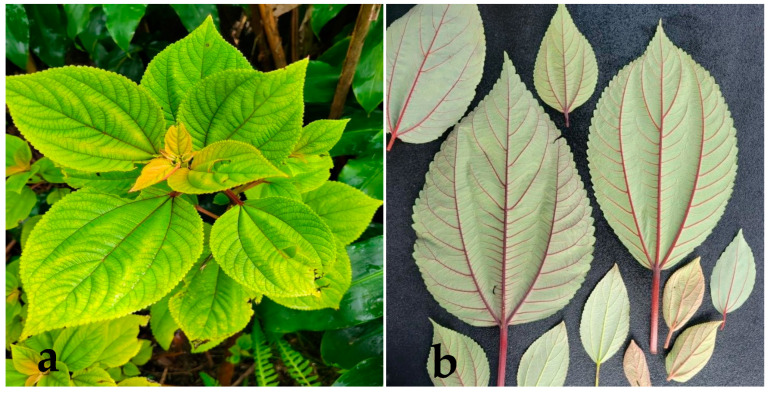
Photographic representation of a māmaki (*P. albidus*) plant specimen, (**a**) Green māmaki, (**b**) Red vein coloration of māmaki. Courtesy of Grant.

A 1992 report by the United States Fish and Wildlife Service estimated that Hawaii is home to over 1200 vascular plant species, many of which are endemic due to the Hawaiian Islands’ geographical isolation and unique biodiversity [1]. Understanding the anthropological and ecological contexts of life on the Hawaiian Islands necessitates special consideration of the novel plant and animal species, including the native plants that may hold cultural significance among indigenous populations. One such vascular plant species that fits this criterion is *Pipturus albidus* (Hook & Arn.) A. Gray, also known as Waimea or, most commonly, as māmaki (Figure 1).

It is important to acknowledge that historically and presently, the common name māmaki has served as an inclusive term encompassing all four species of the *Pipturus* genus native to the Hawaiian Islands [2]. Within the Urticaceae family, the *Pipturus* genus comprises twenty-nine accepted species as of 2023, as documented by the Kew Royal Botanical Gardens online plant database, predominantly inhabiting tropical and subtropical climates across Asia and Pacific Basin islands [3]. Four species of the *Pipturus* genus, namely *P. forbesii*, *P. kauaiensis*, *P. ruber*, *and P. ablidus*, are exclusively endemic to Hawaii [2,4]. For the sake of clarity and coherence, this review strictly employs the term māmaki to specifically denote *P. albidus.*

*P. albidus* holds a significant place in the cultural heritage of indigenous Hawaiian communities, its incorporation predating the era of Western scientific classification, with initial reports dating back to exploratory expeditions conducted by British and French explorers in the late 18th and early 19th centuries [2]. Throughout history, *P. albidus* has undergone multiple taxonomic revisions, largely driven by inconsistencies in earlier documentation of Hawaiian plant species [2]. In 1854, botanist Hugh Algernon Weddell formally established the *Pipturus* genus, and the currently accepted species name of *P. albidus* was subsequently introduced in Alphonse Pyramus de Candolle’s work “Prodromus Systematis Naturalis Regni Vegetabilis” [2]. In 1999, Wagner et al. identified previously recognized *Pipturus* species as being synonymous with *P. albidus* and further revised the genus to the currently accepted four members endemic to the Hawaiian Islands, including *P. albidus* [4].

*P. albidus*, hereafter referred to as māmaki for the purpose of this review, exhibits noteworthy distinction within the *Pipturus* genus by virtue of its enduring utilization by indigenous Hawaiian communities. Besides its application as a material resource, māmaki enjoys recognition for its traditional medicine applications [5,6,7,8]. The claimed health benefits associated with māmaki are diverse, including potential anti-infectious and anti-inflammatory properties [6,8,9]. Over the past decade, growing public awareness of māmaki’s potential therapeutic benefits has spurred increased consumer demand outside of Hawaii, particularly within the United States [2]. This growth in interest has consequently stimulated the development of industries centered around māmaki, including those involved in agriculture, distribution, and retail operations [2]. As consumer interest in māmaki continues to expand, māmaki has the potential to emerge as a distinctive agricultural resource, further facilitating the growth of the commercial industry in Hawaii. Moreover, māmaki participates in significant ecological relationships, such as its involvement in interspecies interactions with vulnerable avian and insect species [10,11,12,13,14].

Despite the cultural, ecological, and medicinal importance of māmaki, many aspects of this plant species remain relatively unexplored. This is, in part, highlighted by the lack of comprehensive literature analysis in published works. Therefore, this review aims to provide an overview of the existing literature on māmaki, covering its therapeutic properties, physical and agricultural properties, nutritional and mineral composition, and active phytochemicals, with a particular focus on areas exploring the potential therapeutic properties of māmaki as it relates to human health. We also highlight recent advances in research on māmaki’s anti-microbial, anti-viral, chemopreventive, anti-inflammatory, and antioxidant activities. Overall, this review aims to foster heightened awareness and understanding of this captivating plant by shedding light on areas of interest.

## 2. Physical Characteristics of Māmaki

Māmaki was characterized by the renowned Swedish botanist Carl Skottsberg in 1934, describing māmaki as “a shrub or rarely small tree with somewhat hairy stems. The phenotypic variation in functional leaf traits of the māmaki is ovate in shape, light green on the upper side and almost white, with very fine short hairs on the underside. The māmaki plant possesses three main veins, often characterized as bright red or purple in color”. [15]. Skottsberg’s description, while encompassing a wide range of variable characteristics, can be considered a reliable and overarching general assessment of the physical attributes exhibited by māmaki.

Māmaki, as a species, displays a considerable degree of phenotypic variation [2,7]. This substantial variability and inconsistencies in the documentation have historically resulted in the classification of māmaki as nine distinct species within the *Pipturus* genus [2]. However, more recent taxonomic revisions have consolidated these variations into a single species, *P. albidus* [2]. The extensive range of phenotypic variation in māmaki poses challenges in interspecies and intraspecies classification, mainly due to overlapping characteristics with other local plant species [2,7]. These phenotypic differences serve as a basis for categorizing māmaki into its noted variants and subvariants. These distinctions are commonly determined by leaf and vein color, typically reflected by the variant’s common names. Identified māmaki variants include, but are not limited to, green māmaki, panaewa, purple māmaki and associated sub-variants, and additional hybrid varieties [7]. Relatively large variations are seen in height, with mature specimens ranging between 2 and 20+ feet, resulting in māmaki being commonly classified as either a shrub or small tree [2,16]. In line with Skottsberg’s approach, the following description of māmaki’s physical characteristics aims to offer a general portrayal of the species as a whole and does not attempt to go into intricate detail regarding the multiple variants that have been recorded.

In a 1989 report published in the Agricultural Handbook no. 679 by Little & Skolmen, māmaki’s outer bark is described as exhibiting a light brown coloration, accompanied by a surface adorned with randomly distributed raised dots. The inner bark of māmaki is characterized by green-colored streaks, possessing a fibrous and mucilaginous texture [17]. The sapwood exhibits a pale, whitish-brown hue, while the soft heartwood retains a reddish-brown palette [17]. The trunk size of māmaki is largely variable, depending on life stage and environmental conditions; however, larger mature specimens of twenty-plus feet in height are noted as having a trunk size of approximately one foot in diameter [17]. The extension of branches and stems from the trunk occurs at protruding nodes, typically arranged in a zig-zag pattern across the bark [17]. Branches are relatively slender and long with respect to the trunk, giving them a notable drooping appearance [18,19]. New growths in the form of twigs may display fine gray hairs, which are similarly noted on the underside of māmaki’s leaves [17]. The root system of māmaki is fine and fibrous, and the sap is described as having a watery consistency [16,20]. As juveniles, māmaki plants display qualities that are more herbaceous in nature but that transition to more woody phenotypes during maturation [16].

One of the more pertinent identifying features of māmaki leaves (Figure 1) is their oval or elliptical shape, with the margins exhibiting toothed wavy edges [2,17]. The size and shape of the leaves can vary within an individual plant, with alterations in leaf sizes often seen along branches [17]. The green upper surface of māmaki leaves can display a range of hues, ranging from pale to dark greens, that are occasionally accompanied by reddish or purplish undertones [17]. Emerging from the leaf stalk, three prominent veins traverse the blade of each leaf, with the two lateral veins radiating outwards along the side [17]. These veins may exhibit variations in color ranging from light green to greenish shades of red and purple [17]. The underside of the māmaki leaf is layered by fine, pale gray hairs, which are reminiscent of the hairs observed in other nettle species [17]. Unlike certain nettle species, however, māmaki’s hairs do not elicit skin irritation upon contact [21].

Māmaki’s leaf stalks exhibit a range of 2.5–7.5 cm in length, while the leaf blades measure between 6 and 20 cm in length and 3 and 15 cm in width [17]. The leaves are relatively thin, displaying a gentle curvature at the margins that taper into a pointed apex, while a gradual thickening occurs toward the base of the leaf [17]. Close examination reveals the presence of microscopic cystoliths, which are minute carbon-calcium mineral growths dispersed on the leaf surface [17,22]. Cystoliths in other plant species are believed to potentially play a role in regulating physiological calcium concentrations and may serve as reservoirs for releasing CO_2_ and H_2_O upon decomposition; whether this is applicable to māmaki has not been clarified [17,22]. In terms of texture, māmaki leaves have been described as ranging from leathery to paper-like, with a subtle roughness to the touch [15,17].

As a monoecious organism, mature individuals of māmaki possess the ability to produce both male and female flowers simultaneously [17]. These flowers, characterized by their greenish-white coloration, are present in clusters that lack stalks and are segregated by sex located on branches in proximity to leaf stems [17]. Māmaki flower clusters exhibit a rounded shape and measure between 6 and 13 mm in diameter, with the sessile female flowers being relatively larger as compared to the males [17]. Māmaki exhibits the ability to continuously fruit throughout the year following maturation [23]. The fruits of māmaki develop as clustered, irregular spheres, displaying a white to translucent appearance, and have an average diameter of approximately 13 mm [17]. Each fruiting body contains individual elliptically shaped seed measuring around 1.5 mm in length [17]. The flavor profile of māmaki’s fruits is commonly described as mild, slightly sweet, or sometimes tasteless [20]. Furthermore, the fruits are adorned with fine hairs that may vary in color, ranging from yellow to pale brown [20].

## 3. Ecological Characteristics of Māmaki

With the exception of Kaho’olawe and Ni’ihau, māmaki is distributed throughout the Hawaiian Islands, growing at elevations reaching up to 6000 feet above sea level [17]. This plant species displays moderate hardiness and can tolerate diverse environmental conditions [4,17,24]. Māmaki predominantly thrives in coastal mesic, lowland mesic, mixed mesic, and wet forest biomes of the Hawaiian Islands, with the availability of moisture, influenced by rainfall patterns and soil retention, being a primary factor governing its geographical range [4,16,24]. Other factors contributing to its distribution include sunlight exposure, temperature, and intra- and interspecies competition [24]. Exhibiting r-type traits, māmaki demonstrates the capacity to colonize disturbed areas rapidly, facilitated in part by a wide seed dispersal range [25]. Māmaki can also establish itself in volcanic environments, showcasing its resilience in recovering from lava-induced wildfires [26,27].

While tolerating a range of environmental conditions, māmaki prefers well-drained, moist soils [16,24]. In soil with inadequate moisture, māmaki is susceptible to rapid desiccation. In contrast, excessively wet soils with poor drainage can lead to root rot and increased vulnerability to pathogens [24]. Māmaki displays adaptability to different soil types, including clay, organic, and volcanic cinder-dominated soils, with a favorable response to higher soil nitrogen concentrations [24]. Although capable of growth in direct sunlight, māmaki is naturally inclined toward forested understory environments, with partial shade conducive to optimal growth conditions [16,24]. Māmaki seedlings have been observed to exhibit rapid germination in areas with canopy gaps but also display viability regarding other canopy configurations, demonstrating flexibility in response to light availability [28,29].

Regarding interspecies ecological relationships, māmaki has been recognized for attracting diverse insect and bird species [10,11,30]. The entomological papers published by Otto Sweezy in 1904 documented the association of māmaki with over fifty insect species. Notably, māmaki readily attracts the only two native butterfly species endemic to the Hawaiian Islands, namely the Kamehameha butterfly (*Vanessa tameamea*) and Hawaiian Blue butterfly (*Udara blackburni*), whose offspring rely on māmaki as a source of shelter and nourishment [14,31]. Moreover, the endemic moth *Udea stellata* utilizes māmaki as a protective habitat for its larval offspring [13]. Young or weakened māmaki plants are susceptible to infestations by various agricultural pests such as twig borers, multiple ant species, termites, and aphids, in addition to fungal infections by *Rhizoctonia*, *Phytophthora*, and *Pucciniastrum boehmeriae* [24,32]. Invasive species have also been observed to be involved in ecological interactions with māmaki. Invasive gastropod herbivores, such as Cuban slugs (*Veronicella cubensis*) and Giant African snails (*Achatina fulica*), pose a threat to māmaki due to their voracious consumption of seedlings [33]. More recently, in 2018, the introduction of the invasive moth species *Arcte coerula* resulted in the defoliation of māmaki [34]. While māmaki itself is not considered threatened, these examples of invasive species predation underscore the potential risks they pose to Hawaii’s endemic species. Additionally, māmaki serves as a habitat and food source for numerous endemic avian species, including those of high conservation concern, such as the Hawaiian thrush (*Myadestes obscurus*), Hawaiian Elepaio (*Chasiempis sandwichensis*), and the endangered Hawaiian crow (*Corvus hawaiiensis*) [10,12,30].

While the interactions between māmaki and nonnative and native animal species have been more readily documented, interspecies plant interactions and competition involving māmaki remain relatively understudied, particularly in agricultural contexts [16,24]. It has been suggested that *Pipturus* species exhibit resistance to competition-induced stress, likely attributable to their regular occurrence in densely populated understory environments alongside native and invasive plant species [35]. A study investigating māmaki’s response to competition with breadfruit trees (*Artocarpus altilis*) in agricultural settings revealed an overall increase in māmaki’s biomass, possibly due to aggressive resource acquisition strategies facilitated by root system expansion [16]. However, the applicability of this aggressive resource allocation model across all māmaki habitats and its implications for agricultural operations are still largely unknown. Further investigations are required to elucidate māmaki’s interspecies interactions, particularly given the predicted future trends of increasing agricultural propagation and the introduction of alien species. It is also essential to recognize that māmaki’s response to anticipated ecological changes resulting from climate change remains understudied.

## 4. Agricultural Propagation, Challenges, and Commercial Industry

The agricultural propagation of māmaki represents a small-scale commercial industry that has experienced a notable upsurge in consumer interest and demand for māmaki tea in recent years [24,36]. Primarily cultivated for its tea leaves, the māmaki agricultural sector caters to local consumption and export markets [24]. Like all commercial crops, certain considerations must be made to mitigate obstacles in the māmaki cultivation process, such as a high juvenile mortality rate [24]. Once successfully established and reaching maturity, māmaki plants can be harvested for multiple years with minimal maintenance requirements before replacement becomes necessary [24].

Māmaki can be agriculturally propagated through both seed and cutting methodologies [24]. Seed propagation generally yields higher success rates, although it may not produce a parental clone, which may be preferable in certain agricultural scenarios [24]. If retainment of parental characteristics is desired, māmaki clones can be easily generated using leaf and hardwood cuttings [24].

As outlined by Sugano et al. in their 2018 report, māmaki seeds can be planted intact or crushed and sprinkled onto selected soil mediums [24]. Direct-to-ground-soil seed propagation demonstrates limited success, while container-based planting with moist mediums has proven more effective in inducing germination [24]. Germination of seedlings typically occurs within a timeframe of 2 to 4 weeks, after which the young plants are transplanted into individual containers. As the individual māmaki plants grow, they are successfully transplanted into progressively larger containers to prevent growth constriction and development inhibition. Finally, once the propagated māmaki plants attain an adequate size, they will undergo a final transition to ground soils [24].

Best practices for the cutting propagation of māmaki adhere to a similar transitional container-based growth methodology, as Sugano et al. (2018) recommended [24]. Generally, isolated māmaki cuttings are first established in containers with moist mediums to stimulate initial growth. As the cutting continues to develop, it is serially planted into larger containers until a target size is reached, where it will then be deposited in agricultural ground soils for long-term cultivation. Different types of parental plant cuttings are reported to exhibit varying degrees of viability, with hardwood cuttings of māmaki showing higher establishment rates than leaf-tip cuttings [24].

Under optimal environmental conditions, māmaki exhibits a relatively rapid growth rate, allowing for the harvest of mature leaves as early as 2.5 to 4 months following ground soil transplantation [24,36]. Subsequent harvests of māmaki leaves can be conducted at intervals of every 2 to 3 months, extending throughout 2 to 3 years before the need for crop replacement arises [24]. Alternative sources propose that plant productivity can be sustained for up to 5 years, permitting extended harvesting periods [36]. Plant replacement requirements are typically driven by a decline in plant vigor resulting from successive leaf harvests [24]. Once an initial crop of māmaki has been established, agricultural operations hold promise for long-term harvesting potential, benefiting from the species’ vigorous growth and the possibility of both vegetative and reproductive propagation throughout a plant’s agricultural lifespan [24,36].

Māmaki agricultural propagation is accompanied by various challenges that can hinder cultivation efforts. These obstacles encompass several aspects, including the tendency of young plants to exhibit root circularization and constriction, transplantation mortality resulting from physical damage and potential pathogen exposure, susceptibility to root rot in excessively moist and poorly drained soils, the possible loss of vigor due to environmental stressors, and the vulnerability of young or weakened plants to pest infestations [24]. To mitigate issues of root circularization, the utilization of elongated cones or rectangular containers is recommended, as these provide ample space, promoting vertical root growth [24]. While effective in stimulating growth, pruning practices must be executed with caution to minimize the risk of pest infestations [24]. Partial shade is advised to optimize growth conditions for young plants, as reflected in māmaki’s natural inclination to thrive in understory environments [24]. However, this preference for partial shade poses challenges for māmaki agricultural operations in lowland, non-forested areas where little to no natural barriers exist to filter sunlight [24]. As demand for māmaki leaves continues to rise, the expansion of māmaki farming into non-forested or suboptimal environments has become increasingly prevalent [24].

Innovative paired cropping techniques are currently under exploration to address the need for more efficient and cost-effective methods of cultivating māmaki beyond its natural range. In 2018, Sugano et al., in collaboration with the University of Hawaii, aimed to investigate an alternative agroforestry cropping system that would enhance the success rate of commercial māmaki farming in the lowlands of Hawaii while minimizing the over-exploitation of māmaki in forested environments [24]. Their study proposed the paired planting of larger shade-producing native Hawaiian plant species to, in turn, generate a more conducive and naturalistic environment for commercial māmaki growth. Although the long-term results of Sugano et al.’s [24] study are still being evaluated, and no subsequent publications have been released, this report sheds light on the commercial challenges associated with an expanding māmaki agricultural industry, given increases in consumer demand.

Similarly to a combined agroforestry approach, another study explored the paired planting of māmaki and breadfruit trees, a practice currently employed by certain Hawaiian agricultural operations [16,36]. Despite some uncertainties, the presence of competition and shade provided by breadfruit trees demonstrated a positive effect on the overall biomass of māmaki plants when paired together [16,36]. While research on māmaki intercropping is relatively nascent, if successfully implemented, these novel approaches have the potential to facilitate intercrop operations within restricted spaces and contribute to cost and resource reduction by promoting a naturalistic ecological setting for māmaki growth, thereby alleviating the need for potentially costly agricultural infrastructure [24].

Despite market growth over the last 7 years, smaller-scale, family-owned farms employing conventional propagation techniques in forested environments continue to be the predominant method of commercial māmaki production [2]. In recognition of this trend, governmental and private entities have shown increased interest in further developing agricultural operations, as seen by the issuance of small business and research grants to expand existing enterprises [2,16]. As of 2022, over 20 brands selling a range of māmaki products are supported by current operations, with loose-leaf tea mixtures and pre-made bottled beverages being the most popular items [2].

## 5. Cultural and Medicinal Use of Māmaki

In the Hawaiian Islands, māmaki has deep cultural significance and has been traditionally utilized by native Hawaiian populations in several applications, including woodworking, textile, and medicinal practices [7,19,20,37]. With its reputation for being easily workable, māmaki hardwood has been employed in tool production [17,38]. A notable tool crafted from māmaki hardwood is the “i’e kuku”, a club used to beat and process bark cloth [5,18]. Bark cloth, which can be partially or entirely derived from the fibrous inner bark of māmaki, is a fundamental material in the historical production of textiles, including clothing, cordage, and paper, by indigenous communities [19,37].

Bark cloth, also referred to as “kapa” in the Hawaiian Islands, is a general term encompassing nonwoven textiles made from various barks, including that of māmaki [39]. Different types of bark cloth exhibit distinct characteristics and applications based on their composition, with māmaki-based bark cloths noted for their strength and durability [37,40]. The prevalence of different types of bark cloth varies by location, with māmaki bark cloths being unique to Hawaii [37,39,40,41]. Hawaiian kapa is incorporated in many products, including clothing such as loin cloths or skirts, netting, cordage, blankets, and artistic creations [39]. Producing bark cloth typically involves peeling the inner bark, moistening it, and flattening it using clubs [39]. Once the desired thickness and shape are achieved, the bark cloth is dried before use [37,39].

Māmaki, as a medicinal resource, was widely consumed by native Hawaiian populations for the treatment of various ailments [7,8,9,15]. Both the leaves and fruits were utilized, with the fruits being consumed directly or applied as poultices, and the leaves brewed into tea [7,18]. Traditional tea preparation involves mixing fresh māmaki leaves into the ground with spring water and hot stones [15,20]. Depending on the specific condition being treated or desired medicinal effects, māmaki could be consumed individually or in combination with other therapeutic substances to achieve desired outcomes [7].

Numerous health benefits have been associated with the consumption of māmaki, and these claims continue to be promoted in contemporary times. The purported health benefits include cholesterol reduction, blood sugar regulation, reductions in blood pressure, cardiovascular benefits, anti-inflammatory effects, stress relief, digestive aid, anti-infectious properties (anti-viral, anti-fungal, and anti-microbial), promotion of liver health, action as a laxative, use as a pregnancy aid, and treatment for thrush and general debility [7,9,15,18,20]. While promising, it is important to note that the majority, if not all, of these health claims lack significant scientific characterization, and the specific active compounds and underlying mechanisms associated remain relatively unknown.

More contemporarily, māmaki is typically consumed as brewed tea produced from dried leaves [20]. One Hawaiian-based retailer, Big Island Coffee Roasters, characterizes māmaki tea as having an earthy and nutty flavor, with a natural sweetness and a lack of bitterness. The steeping time for dried māmaki leaves can vary according to personal preference, with longer steeping resulting in more pronounced flavors. Māmaki tea is often marketed for its perceived health benefits, being advertised as a supplemental treatment for a range of conditions such as diabetes mellitus, depression, rat-lungworm disease, fatigue, and anxiety (Big Island Coffee Roasters, Bee Boys, 2020). Moreover, advertisers often highlight the antioxidant profile of māmaki tea as a selling point (Big Island Coffee Roasters). Given the significance of these claims, it becomes important to conduct investigations to verify the scientific basis for such statements. While preliminary studies indicate potential therapeutic properties, more comprehensive investigations are necessary to validate and fully understand the contexts of māmaki’s medical capabilities.

## 6. Anti-Microbial, Anti-Viral, and Anti-Fungal Activity of Māmaki

Prior data have indicated that māmaki may possess anti-microbial, anti-viral, and anti-fungal properties, as initially demonstrated by Locher et al. in a 1995 report published in the *Journal of Ethnopharmacology* [6]. This study assessed different fractional extracts of māmaki and other Hawaiian medicinal plants for their inhibitory activity against select bacterial, viral, and fungal infectious agents in vitro [6].

Extract-treated disk diffusion assays conducted on Trypticase Soy Agar (TSA) bacterial cultures revealed that māmaki inhibited the growth of two out of the four tested bacterial strains [6]. Specifically, water- and methanol-based extracts derived from the bark and leaves of māmaki, respectively, exhibited growth inhibition against the pathogenic Gram-positive strains *Staphylococcus aureus* and *Streptococcus pyogenes* at a treatment concentration of 100 µg/mL [6]. However, the two Gram-negative bacterial strains, *Escherichia coli* and *Pseudomonas aeruginosa*, demonstrated no discernible response upon exposure to māmaki extracts. Differential inhibitory activity between the bark H_2_O extracts and the leaf methanol māmaki extracts may suggest that the inhibitory active compounds responsible are solvent-specific and distinct. Moreover, differential anti-microbial compositions may be present in the leaves of māmaki as compared to the bark, this being reflected by māmaki leaf H_2_O extracts not exhibiting inhibitory activity against *S. aureus* and *S. pyogenes*, unlike the H_2_O extracts derived from māmaki bark [6]. In the same study, māmaki extracts were also analyzed for their ability to inhibit the growth of three fungal species over a 24 h incubation period at body temperature [6]. In this assessment, māmaki leaf acetonitrile extracts inhibited the growth of *Microsporum canis* and *Trichophyton rubrum* at treatment concentrations of 1000 µg/mL and *Epidermophyton floccosum* at a treatment concentration of 125 µg/mL. These fungal species, known as dermatophytes, have a global distribution and are commonly associated with low-risk dermal infections in humans and other mammals [42,43,44]. While the acetonitrile māmaki leaf extract displayed fungal inhibition, it did not display inhibition against the previously mentioned bacterial strains, suggesting that māmaki leaf’s anti-fungal compounds are distinct from its anti-microbial compounds.

Currently, Locher et al.’s 1995 study represents the first and only published literature investigating the anti-microbial and anti-fungal properties of māmaki [6]. The inhibitory properties demonstrated by māmaki extracts do offer preliminary insight giving initial support to medicinal claims of māmaki’s potential in combating fungal and bacterial infections. Still, the efficacy of these therapies as they relate to established clinical treatments requires further expansion. The possible mechanisms of action and the associated active compounds responsible for the observed growth inhibition in the Gram-positive bacterial strains remain unknown. It remains unclear whether the displayed anti-microbial activity indicates selective inhibition specific toward Gram-positive strains. Similarly, it is unknown whether māmaki would show anti-fungal inhibition of fungal strains outside dermatophytes. This uncertainty highlights a need for follow-up assessments to investigate the broader spectrum of māmaki’s anti-fungal and anti-microbial activity.

Locher et al. (1995) also evaluated the in vitro effects of māmaki extracts on various viruses, including Herpes simplex virus 1 and 2 (HSV 1 and 2), vesicular stomatitis virus (VSV), poliovirus, Semliki Forest virus, and coxsackie B3 virus [6]. The researchers employed a 50% endpoint viral titration technique to compare virus-induced cytopathy between māmaki extract-treated and non-extract-treated cell lines. While not displaying inhibition of poliovirus, Semliki Forest virus, and coxsackie B3, māmaki did exhibit a high degree of anti-viral activity against HSV 1 and 2 and VSV relative to 16 other medicinal plants evaluated in the study. Expressed as the minimal concentration of plant extract needed for the prevention of virus-induced cytopathic effect, the water-based extracts derived from māmaki stems demonstrated inhibition of HSV 1 and 2 at an extract concentration of 250 µg/mL (virus titer of 10^−4^) and VSV at a concentration of 500 µg/mL (virus titer of 10^−4^) [6]. A subsequent 1996 study by Locher et al. further assessed māmaki’s in vitro anti-viral properties by examining its inhibitory activity against human immunodeficiency virus-1 (HIV-1) [8]. By comparing māmaki extract-induced cytotoxicity and HIV-1-induced cytopathy, researchers observed that methanol and water extracts produced from the leaves and bark of māmaki inhibited HIV-1 cytopathy in MT-4 cultured cell lines [8]. In both of Locher et al.’s 1995 and 1996 studies, māmaki extracts demonstrated a relatively low degree of cytotoxicity and a high degree of viral protection, suggesting selectivity toward the viral agents [6,8].

Like māmaki’s anti-fungal and anti-microbial properties, the specific active compounds responsible for the observed anti-viral activity have not been explicitly elucidated in Locher et al.’s 1995 [6] and 1996 [8] studies. Nonetheless, certain assumptions regarding māmaki’s anti-viral compounds can still be inferred from their findings. In Locher et al. (1996), extracts separated māmaki’s compounds based on their polarity [8]. The differential degrees of HIV-1 anti-viral inhibition observed between water and methanol māmaki extracts thus implies the existence of distinct anti-viral components occurring within each respective extract. Additionally, the variances in anti-viral activity observed among extracts derived from different parts of the māmaki plant suggest localized discrepancies in the anti-viral compound composition throughout the plant. This is reflected by the superior levels of anti-viral activity against HIV-1 exhibited by the methanol and water māmaki leaf extracts compared to their stem extract counterparts. Among the many questions raised by Locher et al.’s 1995 and 1996 reports [6,8], the reasons why māmaki extracts’ anti-viral compounds displayed selectivity toward only HSV 1 and 2, VSV, and HIV-1 and not the other viruses evaluated is unclear. Whether this specificity of māmaki’s anti-viral activity towards these viruses is due to similarities in mechanisms of anti-viral action or other factors is unknown.

Overall, little to no additional data exploring the potential anti-infectious activity of māmaki have been published since Locher et al.’s 1995 and 1996 reports [6,8]. Although the results from these studies show promising preliminary evidence, further extensive research is required to understand the scope and capabilities of māmaki as an anti-infectious therapeutic, especially in comparison to other, better understood clinical treatments. Given the growing concern of antibiotic resistance and the continuous evolution of viral strains, there is an increasing need to identify future pharmaceutical compounds that may hold potential as novel or alternative drug therapies against infectious agents. Underrepresented plants used in traditional medicine, such as māmaki, may serve as valuable sources for discovering such compounds. Given the current data, comprehensive investigations are necessary to explore māmaki further and unveil its anti-infectious active compounds and mechanisms of action.

## 7. Chemopreventive and Anti-Inflammatory Activity of Māmaki Leaves and Extracts

Until recently, the investigation of māmaki’s anti-inflammatory and chemopreventive properties remained an underexplored area of research. Presently, the only available literature on this subject is a 2022 study by Sun et al. published in Sage Journal [9]. In this study (Table 1), the authors examined the impact of ethanol- and water-based māmaki extracts on cellular proinflammatory NF-κB pathway signaling and nitrous oxide (NO) production [9]. Additionally, researchers assessed the cytotoxicity of māmaki extracts against lung (LU-1) and breast (MCF-1) cancer cell lines. To prepare the extracts, researchers collected māmaki leaves to produce freeze-dried and dehydrated samples. Samples were then powdered or left as loose-leaf during extraction to determine how different leaf preparations may influence māmaki’s anti-inflammatory, anti-cancer, and chemopreventive properties. Additionally, water-based māmaki extracts were prepared at 27 °C, 60 °C, or 100 °C to simulate different brewing temperatures, while all ethanol-based extracts were prepared at 27 °C [9].

The NF-κB pathway is a proinflammatory signaling pathway implicated in inflammatory diseases and the development of specific cancers, including breast and colon [45,46]. To determine māmaki’s effect on NF-κB signaling, Sun et al. (2022) employed an NF-κB luciferase assay conducted using a stably transfected human embryonic kidney cell 293 reporter line [9]. Analysis revealed that powdered dehydrated māmaki leaf H_2_O extracts prepared at 27 °C, 60 °C, and 100 °C resulted in significant inhibition of NF-κB signaling at 73.7%, 62.7%, and 75%, respectively. Other extracts that produced notable NF-κB inhibition of over 60% include powdered freeze-dried māmaki leaf extracts prepared in 100% ethanol, freeze-dried loose-leaf māmaki H_2_O extracts prepared at 27 °C, and dehydrated loose-leaf māmaki extracts prepared in 70% ethanol [9]. Across all preparation variables (freeze-dried vs. dehydrated, powdered vs. loose-leaf, and temperature), 70% ethanol māmaki extracts demonstrated the highest NF-κB inhibition with an average of 53.0% as compared to H_2_O extracts and 100% ethanol extracts at 48.7% and 48.3%, respectively.

NO production was induced through LPS stimulation in RAW 264.7 macrophages, an immortalized cell line derived from a virus-induced male mouse leukemia. As a mediator molecule involved in a myriad of intercellular signaling events, endogenous NO production can elicit proinflammatory and anti-inflammatory responses depending on concentration and location within the body [47]. In both mice and humans, select scenarios of NO overproduction, as seen in the macrophage immune response, have been noted to induce inflammation and, like NF-κB, are thought to play a role in the pathophysiology of tumor formation in select cancers and cardiovascular diseases such as atherosclerosis [47,48,49,50,51,52]. Due to NO’s instability and short half-life, Sun et al. (2022) determined cellular nitrite concentrations, a metabolite of NO, to indirectly quantify associated cellular NO concentrations [9]. All māmaki samples analyzed demonstrated a NO production inhibition below 60% [9]. An H_2_O extract prepared from freeze-dried powdered māmaki leaves at 27 °C displayed the most potent inhibition of all samples tested, at 55.5%. Ethanol māmaki extracts, namely dehydrated and freeze-dried loose-leaf extracts prepared in 70% or 100% ethanol, also showed NO production inhibition above 50%. Similarly to NF-κB inhibition, the 70% ethanol māmaki extracts were found to have the highest average NO inhibition of 42.3% across all preparation variables. In comparison, 100% ethanol extracts were observed to have an inhibition of 38.7%, and H_2_O extracts of 27.8%.

While it is difficult to make definite statements regarding the solubility profile of the active compounds responsible for both NO and NF-κB inhibition in Sun et al.’s 2022 study, the data do suggest that preparation methods for māmaki leaf extracts could play a role in inhibitory active compound bioavailability [9]. For NF-κB signaling, the highest consistent inhibition was displayed by dehydrated powdered H_2_O extracts across all preparation temperatures (27 °C, 60 °C, 100 °C). This may imply that the dehydration processing and the powdered texture associated with these samples increase the availability of māmaki’s anti-inflammatory compounds related to NF-κB signal transduction in human embryonic reporter cell lines. While speculative, such trends in Sun et al.’s (2022) data may apply to optimizing the māmaki tea leaf extract preparation and brewing for active compound identification and maximum anti-inflammatory benefit [9]. Cytotoxicity assays utilizing lung cancer (LU-1) and breast cancer cell lines (MCF-7) demonstrated that māmaki extracts do not have significant cytotoxic selectivity against cancerous cells. Due to this, it is stated that the potential chemopreventive activity of māmaki through NO and NF-κB inhibition is not correlated with cancer cell cytotoxicity.

Overall, the effect of māmaki extracts on NF-κB and NO proinflammatory activity, as demonstrated by Sun et al. (2022), may provide insight into potential chemopreventive properties and potential anti-inflammatory characteristics, as both have a relationship with proinflammatory signaling and carcinogenic activity [9]. Sun et al.’s (2022) [9] initial results are promising and may help support some health claims surrounding māmaki as a naturopathic therapeutic. However, knowledge regarding the anti-inflammatory and chemopreventive properties of māmaki remains very limited, as both associated mechanisms of action and active compounds involved remain unknown. These findings only emphasize the importance of further research to clarify the possible complex mechanisms that lend themselves to māmaki’s traditional medicinal uses.

## 8. Nutrient and Mineral Profile of Dried Māmaki Leaves

In a 2011 study conducted by Kartika et al., findings revealed that the nutritional and mineral composition of dried māmaki leaves exhibited variations influenced by the season of leaf harvest [7]. Out of the eight macronutrients analyzed, five macronutrients, namely crude protein, acid detergent fiber, neutral detergent fiber, crude fat, and lignin, exhibited significantly different concentrations in dried māmaki leaves between the winter and summer seasons [7]. Specifically, dried māmaki leaves harvested in winter had higher levels of neutral detergent fiber and crude protein than those harvested in summer. Conversely, dried māmaki leaves from summer harvests demonstrated higher concentrations of crude fat, acid detergent fiber, and lignin than in winter samples. While not showing seasonal variation, the other macronutrients analyzed were found at measurable levels and included dry matter, ash, and cellulose. The average concentrations of all macronutrients over both winter and summer seasons were as follows: dry matter (866.1 ± 3.1 mg/g), ash (127.9 ± 9.4 mg/g), crude protein (108.3 ± 35.6 mg/g), crude fat (30.2 ± 7.5 mg/g), neutral detergent fiber (279.6 ± 43.9 mg/g), acid detergent fiber (198.4 ± 20.7 mg/g), lignin 117.2 ± 9.5 mg/g, and cellulose (49.0 ± 10.0 mg/g) [7].

Furthermore, the study compared the nutritional content of dried māmaki leaves with that of other commercial teas, specifically Lipton and *C. sinensis* tea leaves. The macronutrient profile of dried māmaki leaves exhibited similar concentrations of protein, fat, and fiber compared to commercial teas. More specifically, māmaki and *C. sinensis* contained similar amounts of crude protein. In contrast, Lipton commercial tea leaves had a significantly lower protein concentration. Differences were observed in ash content, with māmaki demonstrating an ash concentration more than twice that of both commercial teas.

Kartika et al. (2011) also assessed the mineral content of dried māmaki leaves, categorized as macro-minerals and micro-minerals [7]. The macro-minerals detected in dried māmaki leaves included phosphorus (avg. 1.2 ± 0.4 mg/g), potassium (avg. 9.9 ± 2.2 mg/g), calcium (avg. 36.6 ± 3.2 mg/g), magnesium (avg. 2.5 ± 1.1 mg/g), and sodium (avg. 0.3 ± 0.1 mg/g). Apart from potassium and calcium, significant differences in macro-mineral concentrations were recorded between dried leaves harvested in the winter and summer. Higher concentrations of phosphorus and magnesium were found in winter-harvested samples. In contrast, summer samples demonstrated a higher concentration of sodium. Compared to other commercially available tea leaves such as echinacea, peppermint, red raspberry, and Chinese green teas, dried māmaki leaves displayed significantly higher amounts of calcium but lower potassium, phosphorus, sodium, and magnesium levels.

The micro-mineral content of dried māmaki leaves included boron (avg. 35.7 ± 7.1 µg/g), copper (avg. 8.3 ± 5.3 µg/g), iron (avg. 49.6 ± 5.7 µg/g), manganese (avg. 68.5 ± 29.0 µg/g), molybdenum (avg. 0.8 ± 0.4 µg/g), and zinc (avg. 11.7 ± 2.6 µg/g). Like the macro-minerals, all micro-minerals, except molybdenum, exhibited significant variation between seasons. Copper, iron, manganese, and zinc were found at higher concentrations in the winter season samples. Boron was found at higher concentrations in the summer-harvested dried leaf samples.

While it is challenging to determine the tangible nutritional effects of the nutrient and mineral content variations observed in māmaki leaves between the summer and winter seasons, it should be noted that the overall nutritional significance is relatively low. Direct ingestion of māmaki leaves by consumers remains uncommon. Instead, when considering the nutritional effects of māmaki, the nutritional context of māmaki tea infusions assumes greater significance, as they represent the primary form of consumption. The nutritional content of māmaki leaves is a reference point for comparisons with māmaki tea, enabling an assessment of nutrient and compound transfer efficiency. Consequently, additional research is warranted to explore how variations in the nutritional components of māmaki leaves influence the downstream health benefits of māmaki tea consumption.

## 9. Nutritional and Mineral Content of Māmaki Dried Leaf Tea Infusions

Kartika et al.’s 2011 study examined the nutritional and mineral content of dried māmaki leaves and reported on the nutritional composition of māmaki tea infusions [7]. The harvest season (summer vs. winter) and the specific māmaki tea variants used influenced the tea’s composition. The māmaki variants investigated in this portion of the study included full green, hybrid purple, two purple subvariants, and an agriculturally cultivated hybrid with light pink veins and green leaves known as “panaewa”. Kartika et al. (2011) also investigated how different dried leaf preparation methods could affect the mineral content of māmaki teas [7]. To investigate this, a single māmaki variant, hybrid purple māmaki, was brewed using crushed, ground, or whole dried leaves, and the mineral concentrations were subsequently compared between these preparations.

Seasonal variation in the overall mineral content of māmaki tea infusions followed similar patterns among all varieties. No significant differences were noted between winter and summer sample tea infusions for macro-minerals, and micro-minerals were only present in trace amounts. Therefore, the micro-mineral content of tea infusions was deemed nutritionally insignificant. Kartika et al. (2011) reported the average concentrations of macro-minerals for both the winter and summer harvest seasons as follows: phosphorus (1.5 ± 0.3 µg/g), potassium (98.1 ± 9.3 µg/g), calcium (103.0 ± 18.5 µg/g), magnesium (31.6 ± 3.2 µg/g), and sodium (69.7 ± 6.8 µg/g) [7]. Compared to other commercial teas (Lipton and *C. sinensis*), māmaki tea infusions exhibited lower phosphorus, sodium, and magnesium concentrations. While the difference was less pronounced, māmaki tea infusions also displayed lower levels of potassium. Still, they had significantly higher concentrations of calcium in comparison to other commercial teas. Statistically significant variations in mineral content were observed among the different dried-leaf tea preparations. Although present in trace amounts, micro-minerals were found in higher concentrations in infusions brewed from ground māmaki leaves. Interestingly, infusions from dried whole leaf and crushed leaf preparations demonstrated higher levels of potassium, phosphorus, calcium, and magnesium than māmaki teas produced from ground and powdered preparations despite larger surface areas. The authors suggest that these differences may be attributed to the metabolic roles of minerals in cellular and plant metabolism, influencing their availability to be leached into tea infusions during brewing.

Presently, Kartika et al.’s 2011 report is the only one available that analyzes the nutrient and mineral composition of dried māmaki leaves and tea infusions [7]. While most nutrients and macro-minerals found in māmaki tea infusions are comparable to or lower than their counterparts in other commercial teas, the data presented regarding seasonal variation and tea leaf preparation offer early insights into potential variables that can be manipulated to achieve māmaki tea infusions with higher macro-mineral concentrations and nutritional value.

Further research should also consider the growing conditions of the plants, as variations in soil composition can impact both the leaf and infusion content, potentially leading to more substantial differences. As māmaki is already commonly advertised for its health benefits, a comprehensive understanding of the various factors influencing its nutritional composition can be utilized to generate a product with enhanced commercial value. Such knowledge empowers marketers to endorse a product more attuned to nutritional considerations, potentially elevating its market appeal by being more discerningly aligned with consumer health preferences.

## 10. Antioxidant Content of Māmaki Leaves and Infusions

Māmaki tea is frequently promoted for its rich abundance of antioxidants. However, due to the limited information available, māmaki’s antioxidant profile and its association with the purported health benefits of māmaki are yet to be fully characterized. Two published studies, Kartika et al. (2007) and Sun et al. (2022), have examined the antioxidant properties of māmaki, assessing the polyphenol content and total antioxidant activity of māmaki leaves and tea infusions [9,15].

Antioxidants play crucial roles in protecting cells against oxidative damage induced by free radicals, encompassing reactive oxygen species (ROS) [53]. Dietary sources such as fruits, vegetables, and nuts, as well as endogenous production of antioxidants within the body, serve as the primary means of acquiring these beneficial compounds [54]. Previous investigations have suggested a correlation between diets rich in antioxidants and reduced occurrences of diseases associated with oxidative stress [55]. Polyphenols are a diverse class of phytochemicals acting as secondary metabolites in plants and are involved in an array of significant biological activities [55]. In addition to influencing the physical attributes of plants, such as flavor, color, and taste, many polyphenols are also recognized for their potent antioxidant activity and potential health benefits, including anti-inflammatory and tissue-protectant properties [55]. Being an overarching phytochemical classification, polyphenols include flavonoids, lignans, stilbenes, and phenolic acids [56].

Kartika et al. (2007) examined the polyphenol composition of extracts from fresh māmaki leaves and the total antioxidant activity of māmaki tea infusions [15]. Using aqueous methanol leaf extracts and liquid chromatography-mass spectrometry techniques, researchers identified three major groups of polyphenolic compounds present in māmaki leaves: (+)-catechin, a flavanol, rutin, a flavonoid glycoside, and chlorogenic acid, a phenolic acid [57,58,59]. In addition to antioxidant activity, these three polyphenols have been documented to have potential health benefits, such as anti-obesity, anti-infective, anti-carcinogenic, cardioprotective, and hepatoprotective effects [57,58,59].

The concentrations of (+)catechin, rutin, and chlorogenic acid were compared among H_2_O extracts sourced from the fresh leaves of green, purple, and hybrid purple māmaki [15]. The highest concentrations were observed in extracts from purple māmaki, with a (+)-catechin concentration of 5.0 ± 0.8 mg/g, a chlorogenic acid concentration of 1.7 ± 0.1 mg/g, and a rutin concentration of 1.8 ± 0.1 g/mg. Green māmaki contained the lowest concentrations of (+)-catechins, chlorogenic acid, and rutin at 2.4 ± 0.0 mg/g, 1.1 ± 0.1 mg/g, and 1.1 ± 0.2 mg/g, respectively. The average concentration of chlorogenic acid across all variants was determined to be 1.4 mg/g, while the average (+)-catechin concentration was 3.3 mg/g, and the average rutin concentration was 1.5 mg/g [15].

In comparison to other commercial tea leaves (Gyokuro green, Chinese oolong, and Kenyan black tea leaves), māmaki leaf aqueous extracts displayed significantly higher concentrations of (+)-catechins and rutin [15]. However, as cited by Kartika et al. (2007), comparing chlorogenic acid between māmaki leaves and other commercial teas was not feasible, as the comparative commercial tea samples lacked chlorogenic acid concentrations [15]. Rather, chlorogenic acids are more readily found in root vegetables and other select fruits, such as radishes and blueberries, which were not used for comparison in the study [15]. The uncommon presence of chlorogenic acid in māmaki leaves distinguishes it from the other tea species evaluated in the study.

Kartika et al. (2007) further analyzed the seasonal TAA of dried leaf māmaki tea infusions produced from the green, purple, and hybrid purple varieties through photochemiluminescence methodologies, expressing TAA in ascorbic acid equivalents [15]. Results indicated no significant differences in the TAA of māmaki tea infusions brewed from leaves harvested during the winter and summer seasons. Moreover, similarities were observed in the TAA content of māmaki tea infusions produced from the different varieties evaluated. The average TAA values were determined to be 238 ± 23 mg AA/g for purple māmaki, 244 ± 10 mg AA/g for hybrid purple māmaki, and 259 ± 67 mg AA/g for green māmaki [15]. Relative to commercial teas, the average TAA of māmaki tea infusions was similar to that of green teas but significantly higher than that of oolong and black teas. Lastly, Kartika et al. (2007) determined the temporal trends of TAA in māmaki tea infusions over 72 h [15]. Researchers found that the TAA of purple māmaki tea infusions decreased over time, with the highest activity being recorded immediately after brewing (293 ± 145 mg AA/g) and the lowest activity at 72 h (163 ± 18 mg AA/g) [15]. The authors noted that eventual stabilization of TAA occurs following 48 to 72 h at refrigerated temperatures.

Sun et al.’s 2022 report also assessed the TAA of māmaki aqueous extracts. The antioxidant activity of dried-leaf māmaki extracts was measured through ferric reducing antioxidant power (FRAP) assays and expressed as μM/μg [9]. Contrasted with juiced cucumber, lilikoi, lemon, and papaya samples, the TAA of māmaki tea infusions revealed significantly higher FRAP values of 40.0 μM/μg, exceeding the antioxidant activity of the comparative samples by up to 40 times. The data presented in both Kartika et al. (2007) and Sun et al.’s (2022) studies suggest that māmaki teas contain relatively high concentrations of antioxidants compared to other commercial teas and select fruits and vegetables [9,15]. The similarities in TAA that māmaki tea shares with green tea, as presented in Kartika et al. (2007) [15], are particularly notable. Green tea consumption has long been associated with various health benefits, including some that mirror the purported health claims surrounding māmaki, like favorable blood sugar regulation and the promotion of cardiovascular health [60]. Green tea’s health benefits are thought to be in part tied to its rich antioxidant profile, which includes beneficial phenolic compounds like (−)-epigallocatechin-3-gallate, a catechin [60]. Similarly, other herbal teas such as yerba mate (*Ilex paraguariensis*) have also been proposed to have a range of health benefits attributed largely to their polyphenol content. Yerba mate leaves have been found to be a source of chlorogenic acids and, like māmaki, yerba mate consumption has been associated with blood sugar regulation, cardioprotective properties, and a reduction in cholesterol [61]. Māmaki’s leaves also contain relatively high levels of polyphenolic compounds, predominantly rutin, (+)-catechins, and chlorogenic acid. Although māmaki tea leaves and infusions likely differ in their antioxidant and polyphenol compositions relative to green tea and yerba mate, comparable mechanisms of antioxidant action due to the presence of phenolic compounds may lead to similar human health outcomes.

Due to differences in TAA assessment, it becomes difficult to directly compare the TAA of māmaki tea infusions between the data presented by Kartika et al. (2007) and Sun et al. (2022) [9,15]. Moreover, because these two works represent the entirety of published data assessing māmaki’s antioxidant composition, no additional literature is present to further confirm their findings, this including the potentially beneficial polyphenol content of māmaki leaves as presented by Kartika et al. (2007) [15]. Further in-depth research is needed to more completely elucidate māmaki’s antioxidant profile, including the plant itself and associated consumer products like māmaki tea. Replication and the expansion of prior studies, further comparing māmaki leaves and tea with other known plants, would give more context to māmaki’s overall antioxidant composition. Additionally, a more comprehensive understanding of the different antioxidant compounds present in māmaki would aid in identifying active compounds and cellular mechanisms of action, as they may contribute to the commonly cited health benefits associated with consumption.

## 11. Future Directions and Conclusions

Māmaki lacks the scientific characterization it deserves, largely due to a lack of public awareness outside of the Hawaiian Islands. This is reflected by gaps in the published literature, despite long-lasting historical claims of māmaki’s potential health benefits, its cultural importance among the native populations, and its associated ecological interactions with other endemic species. However, increases in consumer interest have led to the expansion of māmaki-focused commercial industries, which may stimulate further academic interest. This coincides with surges in “nutraceutical” interest and research, an evolving billion-dollar industry focusing on discovering and using alternative, naturalistic therapeutics to treat a wide range of conditions [62]. As an understudied species, māmaki presents numerous avenues for further investigation, including ecological, agricultural, medicinal, and ethnological directions. While this literature review focuses predominantly on areas of future research related to medicinal applications and their associated mechanisms, it is important to note that this is only a snapshot representation of māmaki’s potential for further scientific characterization.

To make further assessments regarding māmaki’s impact on human health, identifying active compounds within māmaki is paramount. Presently, the bioactive compounds and associated mechanisms of action in māmaki remain largely unknown. However, information about the nutritional and antioxidant content may shed some light on potential compounds that may be involved. Further isolation of compounds in māmaki and māmaki tea infusions through fractionation and other methodologies is needed to facilitate future identification and testing. Laboratory techniques such as mass spectrometry (MS), nuclear magnetic resonance spectrometry (NMR), infrared spectroscopy, and matrix-induced laser desorption-ionization (MALDI) can be utilized to identify compounds present in purified extracts. Compound identification will also provide more insight into potential mechanisms of action related to specific health effects and allow for more direct clinical comparisons with known pharmaceuticals.

Other potential avenues of research into māmaki’s health effects include intracellular-based approaches, where in vitro RNA and DNA sequencing methodologies can be used to evaluate the gene expression of cells in response to māmaki treatments, including mRNA and miRNA sequencing. Expression of specified genes related to select cellular responses, such as proinflammatory responses, can also be quantified through techniques such as qPCR. These processes may aid in identifying proteins and cellular pathways promoted or inhibited by māmaki’s compounds. Similarly, RNA and DNA sequencing can be used on cells present in the māmaki plant, not only to characterize māmaki’s intra- and intercellular activity better but also to give a better understanding of what cellular gene products are present. Māmaki cell sequencing data also can be utilized to provide additional information regarding genetic differences between māmaki variants and how these differences may influence taxonomy, ecology, and human health.

Apart from in vitro approaches, there is a critical lack of in vivo and in silico studies analyzing māmaki. Moving forward, additional in silico and in vivo studies will be needed to further solidify māmaki’s potential health benefits. Additionally, while its long history of use strongly suggests that māmaki is safe for consumption, little information exists on māmaki’s toxicology profile and whether there are any adverse health effects associated with ingestion. To build a more comprehensive understanding of māmaki’s health effects, all aspects must be evaluated, as only then will māmaki’s true medicinal value come to light. Regarding the therapeutic applications of māmaki, the most promising studies center on the possibility of māmaki as an anti-viral, anti-microbial, anti-fungal, and anti-inflammatory agent [6,8,9]. While data are limited, māmaki’s anti-viral activity against HIV-1 replication, as demonstrated by Locher et al. (1996) [8], is of particular note. Though antiretroviral therapies have considerably increased the life expectancies of individuals infected with HIV-1, viral resistance to prescribed pharmaceutical treatments continually poses a risk to affected populations, highlighting a need for potential alternatives that have different mechanisms of anti-viral action [63]. While it is currently speculative to discuss whether māmaki’s anti-viral compounds may have the potential to act as an alternative therapeutic for high-impact diseases like HIV-1, the inhibition of HIV-1 by māmaki extracts in Locher et al. (1996) [8] serves as an example of how medicinal plants utilized in traditional medicine may serve as potential sources for major pharmaceutical compounds. In addition to following up on māmaki as an anti-infective for the viruses and organisms previously documented by Locher et al.’s 1995 and 1996 reports, further exploration should also be made to assess māmaki’s effects on other viruses, bacteria, and fungi [6,8]. For example, māmaki has shown inhibition of Gram-positive pathogenic bacteria and DNA viruses, so evaluating māmaki’s effects on similar infective agents may yield exciting results [6,8].

Early evidence of māmaki’s potential anti-inflammatory and chemopreventive capabilities has been demonstrated by Sun et al.’s (2022) report, where inhibition of the proinflammatory NF-κB signaling and cancer macrophage NO production was shown [9]. These mechanisms represent only two of many different proinflammatory signaling mechanisms occurring in cells throughout the human body. Other major proinflammatory pathways, such as JAK-STAT or MAP-K, should also be evaluated. Like NF-κB, both the JAK-STAT and MAP-K proinflammatory pathways have been associated with a range of chronic inflammatory diseases, like psoriasis and rheumatoid arthritis, and have also been implicated in the formation of cancers [64,65,66]. By comprehending māmaki’s anti-inflammatory activity over a broader spectrum, we can, in turn, infer its effect on select health conditions due to the role inflammation plays in the pathogenesis of various diseases. If māmaki does prove to be an effective anti-inflammatory agent, then this may also lend support to māmaki’s action as an alternative treatment for a range of diseases associated with chronic inflammation, such as atherosclerosis, arthritis, non-alcoholic fatty liver disease, and diabetes [67].

Further characterization of māmaki’s ecology is also justified, especially with concerns regarding a growing agricultural industry, habitat loss, and risks posed by invasive species-induced environmental stress. While information is known about māmaki’s life cycle, its ecological roles, besides being a common understory plant in wet forest ecosystems, are understudied. Its wide range and variation across the islands suggest that māmaki may be capable of adaptation and may encompass different ecological niches. However, the extent of māmaki’s influence in community structures seems relatively unknown outside of select interactions. More detailed depictions of māmaki’s role in community structures may help guide land management policies and practices to restore Hawaiian ecosystems’ original flora and fauna. The widespread nature of the plant also belies the fact that varieties and subspecies of māmaki are potentially undocumented, with further research needed to confirm the distinctions between them.

Māmaki’s intraspecies and interspecies interactions become especially pertinent when considering the intercropping, agroforestry, and environmental restoration projects that are currently being investigated. Specific interactions have been noted between māmaki and critically threatened Hawaiian species, such as the Hawaiian crow and Kamehameha butterfly [12,14]. These species’ populations have been negatively impacted by aggressive invasive organisms’ encroachment and habitat degradation [12,14]. Further assessment of these and similar relationships is important in reinvigorating Hawaiian forested ecosystems’ biodiversity. Overall, understanding the ecological features of māmaki not only assists in creating better commercial practices but also may aid in restoring native environments where it is present.

In conclusion, māmaki holds cultural significance among Native Hawaiian populations. It has been integrated into traditional practices for centuries due to its purported medicinal benefits and material applications. It has been associated with several health benefits, including potential anti-viral, anti-fungal, anti-microbial, and anti-inflammatory properties. The limited research data and the large literature gap related to māmaki, while causing difficulties in assessing the action of māmaki, allow for almost endless avenues for further research. From a cultural, medicinal, and ecological perspective, it is clear that māmaki holds great importance and deserves further exemplification. By promoting māmaki, it is the hope of this review to increase the awareness surrounding this incredible plant, hopefully supporting beneficial economic and medicinal outcomes that serve to better indigenous communities and the Hawaiian Islands as a whole.

## Figures and Tables

**Table 1:** Chemopreventive and anti-inflammatory effects of *P. albidus* (māmaki) extracts on proinflammatory NF-κB signaling (HEK-293 reporter cell lines), macrophage nitric oxide production (RAW 264.7 macrophages), and lung (LU-1)/breast (MCF-1) cancer cell lines in vitro. Modified from Sun, Kondratyuk, Wongwiwatthananukit, Sun and Chang [9].

*P. albidus* (Māmaki) Extract/Infusion (20 µg/mL)	Inhibition of Proinflammatory NF-κB Signaling Pathway (Luciferase Assay; HEK-293 Reporter Cell Line)	Inhibition of Nitric Oxide Formation (Nitrite Assay; Mouse LPS Stimulated RAW 264.7 Macrophage Cells)	Lung Cancer Cells (LU-1) and Breast Cancer Cells (MCF-1) SRB^F^ Cytotoxicity Assay
Powdered Freeze- Dried (FD) Māmaki H_2_O Infusions	H_2_O FD powder infusion prepared at 27 °C resulted in 27.4 ± 10.9% inhibition of NF-κB activity and a HEK-293 percent survival of 82.4 ± 2.6%	H_2_O FD powder infusion prepared at 27 °C resulted in a 55.5 ± 0.4% inhibition of NO and a RAW 264.7 macrophage percent survival of 54.9 ± 16.0%	H_2_O FD powder infusion prepared at 27 °C resulted in a percent survival of 84.2 ± 2.0% of LU-1 and 80.1 ± 7.1% MCF-7 cell lines following a 72 h treatment period
	H_2_O FD powder infusion prepared at 60 °C resulted in 28.9 ± 3.3% inhibition of NF-κB activity and an HEK-293 percent survival of 90.2 ± 5.5%	H_2_O FD powder infusion prepared at 60 °C resulted in a 44.5 ± 10.8% inhibition of NO and a RAW 264.7 macrophage percent survival of 86.6 ± 15.8%	H_2_O FD powder infusion prepared at 60 °C resulted in a percent survival of 95.8 ± 5.0% of LU-1 and 95.2 ± 9.2% MCF-7 cell lines following a 72 h treatment period
	H_2_O FD powder infusion prepared at 100 °C resulted in 54.9 ± 1.0% inhibition of NF-κB activity and an HEK-293 percent survival of 90.6 ± 8.0%	H_2_O FD powder infusion prepared at 100 °C resulted in a 39.0 ± 14.9% inhibition of NO and a RAW 264.7 macrophage percent survival of 96.6 ± 7.4%	H_2_O FD powder infusion prepared at 100 °C resulted in a percent survival of 94.1 ± 8.9% of LU-1 and 96.0 ± 6.1% MCF-7 cell lines following a 72 h treatment period
Powdered Freeze- Dried (FD) Māmaki Ethanol Infusions	70% Ethanol FD powder infusion prepared at 27 °C resulted in 56.9 ± 7.0% inhibition of NF-κB activity and an HEK-293 percent survival of 102.0 ± 9.7%	70% ethanol FD powder infusion prepared at 27 °C resulted in a 38.2 ± 5.6% inhibition of NO and a RAW 264.7 macrophage percent survival of 96.2 ± 9.1%	70% ethanol FD powder infusion prepared at 27 °C resulted in a percent survival of 97.2 ± 8.2% of LU-1 and 97.2 ± 3.1% MCF-7 cell lines following a 72 h treatment period
	100% ethanol FD powder infusion prepared at 27 °C resulted in 61.9 ± 4.4% inhibition of NF-κB activity and an HEK-293 percent survival of 105.3 ± 8.3%	100% ethanol FD powder infusion prepared at 27 °C resulted in a 15.3 ± 17.1% inhibition of NO and a RAW 264.7 macrophage percent survival of 95.0 ± 10.5%	100% ethanol FD powder infusion prepared at 27 °C resulted in a percent survival of 98.5 ± 12.3% of LU-1 and 99.3 ± 7.4% MCF-7 cell lines following a 72 h treatment period
Freeze-Dried (FD) Māmaki Leaf H_2_O Infusions	H_2_O FD leaf infusion prepared at 27 °C resulted in 60.6 ± 3.8% inhibition of NF-κB activity and an HEK-293 percent survival of 88.2 ± 7.0%	H_2_O FD leaf infusion prepared at 27 °C resulted in a 4.7 ± 9.7% inhibition of NO and a RAW 264.7 macrophage percent survival of 88.8 ± 17.7%	H_2_O FD leaf infusion prepared at 27 °C resulted in a percent survival of 99.8 ± 10.2% of LU-1 and 99.2 ± 9.4% MCF-7 cell lines following a 72 h treatment period
	H_2_O FD leaf infusion prepared at 60 °C resulted in 39.4 ± 6.4% inhibition of NF-κB activity and an HEK-293 percent survival of 101.2 ± 11.4%	H_2_O FD leaf infusion prepared at 60 °C resulted in a −1.8 ± 1.1% inhibition of NO and a RAW 264.7 macrophage percent survival of 96.2 ± 13.0%	H_2_O FD leaf infusion prepared at 60 °C resulted in a percent survival of 97.9 ± 11.3% of LU-1 and 99.4 ± 5.8% MCF-7 cell lines following a 72 h treatment period
	H_2_O FD leaf infusion prepared at 100 °C resulted in 59.7 ± 4.8% inhibition of NF-κB activity and an HEK-293 percent survival of 96.0 ± 10.8%	H_2_O FD leaf infusion prepared at 100 °C resulted in a 17.1 ± 7.8% inhibition of NO and a RAW 264.7 macrophage percent survival of 98.1 ± 9.8%	H_2_O FD leaf infusion prepared at 100 °C resulted in a percent survival of 99.4 ± 11.0% of LU-1 and 102.9 ± 2.9% MCF-7 cell lines following a 72 h treatment period
Freeze-Dried (FD) Māmaki Leaf Ethanol Infusions	70% ethanol FD leaf infusion prepared at 27 °C resulted in 58.3 ± 2.8% inhibition of NF-κB activity and an HEK-293 percent survival of 86.4 ± 12.2%	70% ethanol FD leaf infusion prepared at 27 °C resulted in a 36.1 ± 6.3% inhibition of NO and a RAW 264.7 macrophage percent survival of 101.2 ± 6.4%	70% ethanol FD leaf infusion prepared at 27 °C resulted in a percent survival of 92.2 ± 18.1% of LU-1 and 99.0 ± 2.1% MCF-7 cell lines following a 72 h treatment period
	100% ethanol FD leaf infusion prepared at 27 °C resulted in 38.1 ± 12.1% inhibition of NF-κB activity and an HEK-293 percent survival of 81.2 ± 8.0%	100% ethanol FD leaf infusion prepared at 27 °C resulted in a 52.6 ± 6.0% inhibition of NO and a RAW 264.7 macrophage percent survival of 96.8 ± 9.7%	100% ethanol FD leaf infusion prepared at 27 °C resulted in a percent survival of 96.8 ± 11.3% of LU-1 and 102.8 ± 2.7% MCF-7 cell lines following a 72 h treatment period
Powdered Dehydrated Māmaki H_2_O Infusions	H_2_O dehydrated powder infusion prepared at 27 °C resulted in 73.7 ± 6.8% inhibition of NF-κB activity and an HEK-293 percent survival of 55.9 ± 9.5%	H_2_O dehydrated powder infusion prepared at 27 °C resulted in a 32.1 ± 5.2% inhibition of NO and a RAW 264.7 macrophage percent survival of 101.3 ± 3.6%	H_2_O dehydrated powder infusion prepared at 27 °C resulted in a percent survival of 101.5 ± 0.7% of LU-1 and 102.7 ± 5.0% MCF-7 cell lines following a 72 h treatment period
	H_2_O dehydrated powder infusion prepared at 60 °C resulted in 62.7 ± 7.3% inhibition of NF-κB activity and an HEK-293 percent survival of 53.4 ± 2.6%	H_2_O dehydrated powder infusion prepared at 60 °C resulted in a 27.6 ± 12.3% inhibition of NO and a RAW 264.7 macrophage percent survival of 100.1 ± 3.5%	H_2_O dehydrated powder infusion prepared at 60 °C resulted in a percent survival of 102.5 ± 4.1% of LU-1 and 103.8 ± 1.4% MCF-7 cell lines following a 72 h treatment period
	H_2_O dehydrated powder infusion prepared at 100 °C resulted in 75.0 ± 8.4% inhibition of NF-κB activity and an HEK-293 percent survival of 43.3 ± 1.5%	H_2_O dehydrated powder infusion prepared at 100 °C resulted in an 18.4 ± 14.1% inhibition of NO and a RAW 264.7 macrophage percent survival of 102.2 ± 0.9%	H_2_O dehydrated powder infusion prepared at 100 °C resulted in a percent survival of 102.1 ± 2.8% of LU-1 and 105.8 ± 2.8% MCF-7 cell lines following a 72 h treatment period
Powdered Dehydrated Māmaki Ethanol Infusions	70% ethanol dehydrated powder infusion prepared at 27 °C resulted in 28.6 ± 8.4% inhibition of NF-κB activity and an HEK-293 percent survival of 47.4 ± 3.8%	70% ethanol dehydrated powder infusion prepared at 27 °C resulted in a 44.5 ± 10.8% inhibition of NO and a RAW 264.7 macrophage percent survival of 102.5 ± 0.3%	70% ethanol dehydrated powder infusion prepared at 27 °C resulted in a percent survival of 102.8 ± 1.8% of LU-1 and 102.7 ± 3.5% MCF-7 cell lines following a 72 h treatment period
	100% ethanol dehydrated powder infusion prepared at 27 °C resulted in 56.2 ± 1.8% inhibition of NF-κB activity and an HEK-293 percent survival of 45.6 ± 0.0%	100% ethanol dehydrated powder infusion prepared at 27 °C resulted in a 35.0 ± 4.1% inhibition of NO and a RAW 264.7 macrophage percent survival of 103.2 ± 0.3%	100% ethanol dehydrated powder infusion prepared at 27 °C resulted in a percent survival of 106.5 ± 1.9% of LU-1 and 101.9 ± 4.2% MCF-7 cell lines following a 72 h treatment period
Dehydrated Māmaki Leaf H_2_O Infusions	H_2_O dehydrated leaf infusion prepared at 27 °C resulted in 45.3 ± 1.0% inhibition of NF-κB activity and an HEK-293 percent survival of 49.9 ± 8.2%	H_2_O dehydrated leaf infusion prepared at 27 °C resulted in a 30.5 ± 9.7% inhibition of NO and a RAW 264.7 macrophage percent survival of 103.6 ± 0.9%	H_2_O dehydrated leaf infusion prepared at 27 °C resulted in a percent survival of 101.5 ± 3.3% of LU-1 and 102.0 ± 3.7% MCF-7 cell lines following a 72 h treatment period
	H_2_O dehydrated leaf infusion prepared at 60 °C resulted in 3.0 ± 13.1% inhibition of NF-κB activity and an HEK-293 percent survival of 49.4 ± 8.4%	H_2_O dehydrated leaf infusion prepared at 60 °C resulted in a 22.6 ± 5.2% inhibition of NO and a RAW 264.7 macrophage percent survival of 104.1 ± 1.6%	H_2_O dehydrated leaf infusion prepared at 60 °C resulted in a percent survival of 104.2 ± 0.5% of LU-1 and 104.2 ± 3.3% MCF-7 cell lines following a 72 h treatment period
	H_2_O dehydrated leaf infusion prepared at 100 °C resulted in 53.8 ± 11.1% inhibition of NF-κB activity and an HEK-293 percent survival of 56.5 ± 8.1%	H_2_O dehydrated leaf infusion prepared at 100 °C resulted in a 44.7 ± 0.0% inhibition of NO and a RAW 264.7 macrophage percent survival of 105.0 ± 0.2%	H_2_O dehydrated leaf infusion prepared at 100 °C resulted in a percent survival of 103.6 ± 2.6% of LU-1 and 100.5 ± 3.2% MCF-7 cell lines following a 72 h treatment period
Dehydrated Māmaki Leaf Ethanol Infusions	70% ethanol dehydrated leaf infusion prepared at 27 °C resulted in 68.4 ± 3.1% inhibition of NF-κB activity and an HEK-293 percent survival of 96.4 ± 11.5%	70% ethanol dehydrated leaf infusion prepared at 27 °C resulted in a 50.5 ± 4.5% inhibition of NO and a RAW 264.7 macrophage percent survival of 101.7 ± 3.0%	70% ethanol dehydrated leaf infusion prepared at 27 °C resulted in a percent survival of 100.1 ± 1.8% of LU-1 and 95.6 ± 9.1% MCF-7 cell lines following a 72 h treatment period
	100% ethanol dehydrated leaf infusion prepared at 27 °C resulted in 37.0 ± 8.5% inhibition of NF-κB activity and an HEK-293 percent survival of 97.1 ± 9.5%	100% ethanol dehydrated leaf infusion prepared at 27 °C resulted in a 52.1 ± 2.2% inhibition of NO and a RAW 264.7 macrophage percent survival of 70.5 ± 11.1%	100% ethanol dehydrated leaf infusion prepared at 27 °C resulted in a percent survival of 85.1 ± 2.5% of LU-1 and 71.3 ± 1.4% MCF-7 cell lines following a 72 h treatment period

## Data Availability

No new data were created or analyzed in this study. Data sharing is not applicable to this article.
